# Sensitivity of self-reported opioid use in case-control studies: Healthy individuals versus hospitalized patients

**DOI:** 10.1371/journal.pone.0183017

**Published:** 2017-08-30

**Authors:** Hamideh Rashidian, Maryam Hadji, Maryam Marzban, Mahin Gholipour, Afarin Rahimi-Movaghar, Farin Kamangar, Reza Malekzadeh, Elisabete Weiderpass, Abbas Rezaianzadeh, Abdolvahab Moradi, Nima Babhadi-Ashar, Reza Ghiasvand, Hossein Khavari-Daneshvar, Ali Akbar Haghdoost, Kazem Zendehdel

**Affiliations:** 1 Neuroscience Research Center, Institute of Neuropharmacology, Kerman University of Medical Sciences, Kerman, Iran; 2 Cancer Research Center, Cancer Institute of Iran, Tehran University of Medical Sciences, Tehran, Iran; 3 Student’ Research Center Committee, Shiraz University of Medical Science, Shiraz, Iran; 4 Golestan Research Center of Gastroenterology and Hepatology, Golestan University of Medical Sciences, Gorgan, Iran; 5 Iranian National Center for Addiction Studies (INCAS), Tehran University of Medical Sciences, Tehran, Iran; 6 Department of Public Health Analysis, School of Community Health and Policy, Morgan State University, Baltimore, Maryland, United States of America; 7 Digestive Oncology Research Center, Digestive Diseases Research Institute, Tehran University of Medical Sciences, Tehran, Iran; 8 Department of Research, Cancer Registry of Norway, Institute of Population-Based Cancer Research, Oslo, Norway; 9 Department of Community Medicine, Faculty of Health Sciences, University of Tromsø, The Arctic University of Norway, Tromsø, Norway; 10 Department of Medical Epidemiology and Biostatistics, Karolinska Institutet, Stockholm, Sweden; 11 Genetic Epidemiology Group, Folkhälsan Research Center, Helsinki, Finland; 12 Colorectal Research Center, Shiraz University of Medical Sciences, Shiraz, Iran; 13 Oslo Centre for Biostatistics and Epidemiology, Institute of Basic Medical Sciences, University of Oslo, Oslo, Norway; 14 Regional Knowledge Hub, and WHO Collaborating Centre for HIV Surveillance, Institute for Futures Studies in Health, Kerman University of Medical Sciences, Kerman, Iran; 15 Cancer Biology Research Center, Cancer Institute of Iran, Tehran University of Medical Sciences, Tehran, Iran; Howard University, UNITED STATES

## Abstract

**Background:**

Several case-control studies have shown associations between the risk of different cancers and self-reported opium use. Inquiring into relatively sensitive issues, such as the history of drug use, is usually prone to information bias. However, in order to justify the findings of these types of studies, we have to quantify the level of such a negative bias. In current study, we aimed to evaluate sensitivity of self-reported opioid use and suggest suitable types of control groups for case-control studies on opioid use and the risk of cancer.

**Methods:**

In order to compare the validity of the self-reported opioid use, we cross-validated the response of two groups of subjects 1) 178 hospitalized patients and 2) 186 healthy individuals with the results of their tests using urine rapid drug screen (URDS) and thin layer chromatography (TLC). The questioners were asked by trained interviewers to maximize the validity of responses; healthy individuals were selected from the companions of patients in hospitals.

**Results:**

Self-reported regular opioid use was 36.5% in hospitalized patients 19.3% in healthy individuals (p-value> 0.001).The reported frequencies of opioid use in the past 72 hours were 21.4% and 11.8% in hospitalized patients and healthy individuals respectively. Comparing their responses with the results of urine tests showed a sensitivity of 77% and 69% among hospitalized patients and healthy individuals for self-reports (p-value = 0.4). Having corrected based on the mentioned sensitivities; the frequency of opioid regular use was 47% and 28% in hospitalized patients and healthy individuals, respectively. Regular opioid use among hospitalized patients was significantly higher than in healthy individuals (p-value> 0.001).

**Conclusion:**

Our findings showed that the level of opioid use under-reporting in hospitalized patients and healthy individuals was considerable but comparable. In addition, the frequency of regular opioid use among hospitalized patients was significantly higher than that in the general population. Altogether, it seems that, without corrections for these differences and biases, the results of many studies including case-control studies on opioid use might distort findings substantially.

## Introduction

Self-report bias is a major source of error on the estimates of illegal drugs use or other sensitive issues. About 30 to 70 percent of persons with positive urine test results refuse reporting illicit drug use; however, the magnitude of self-report bias depends on the study population [1]. In conducting case-control studies, we should bear in mind that hospitalized patients as a control may be more collaborative than healthy individuals, and they tend to report more on their drug use patterns, leading to less self-report bias [2, 3].

Control selection is an essential issue in case-control studies. The inclusion and exclusion criteria for control selection need to be explicit. In order to select hospitalized patients as controls in case-control studies, they should be free from the disease of interest and their current disease should be unrelated to the exposure of interest [4, 5]. Some studies have shown that hospitalized patients are similar in some of their exposures, and selecting them as controls could bias results towards the null hypothesis [6].

The exposure frequency in controls should be representative of the study population that cases come from [7]. Although population-based controls are one of the choices in the design of a case-control study, it is not feasible in all situations. When studying the association between cancer and opioid use, which is an illegal behavior, population-based controls may underreport their exposure history compared to the cancer patients, leading to differential misclassification and overestimation of the disease-exposure association. According to Abnet etal. study[8], self-reported opioid use is common in the rural and urban populations of Golestan province, located in northeastern region of Iran. Population-based controls are reliable, but in order to find the best control and examine the validity in different populations, more studies are recommended.

In studies comparing drug use self-report with urine analyses, the specificity of drug use self-report was above 90% and the sensitivity varied from 40% to 75% [9]. Therefore, we assume that false positives for drug use self-report are rare and our concern is mostly for false negatives. This study aimed to estimate sensitivity of self-reported opioid use for the past 72 hours and suggest optimal control groups for conducting case-control studies on opioid use as their main exposure. Our results will be useful in conducting case-control studies to evaluate the association between opioid use and the risk of cancer.

## Methods

### The study population

The current study was a cross-sectional study of an ongoing large multicenter case-control study investigating associations between opium use and the risk of different cancers. We selected study subjects from the same four provinces where our case-control study will be conducted, namely Kerman and Fars in the southern region, Golestan in the northeastern region, and Tehran, the capital city of Iran, because the prevalence of opium use is high in these provinces.

We compared 178 hospitalized patients and 186 healthy individuals (companions of patients in hospitals) in terms of opioid use self-report sensitivity for the past 72 hours. Only men were included, as opium use is rare among women [10, 11]. We tried to include participants aged from 30 and 75 years old, to accommodate evidence of self-reporting bias for an older population which is usually recruited in case-control studies on cancer. We excluded people with a history of cancer.

#### Hospitalized patients group

We selected study hospitals and wards using purposive sampling. Hospitals were referral centers for our cancer patients in each province. We selected hospitalized patients with a diagnosis not related to our study’s main exposure of interest (i.e. opium use) and our study hospitals were the main treatment centers for them, so they had approximately similar referral pattern to our cancer patients. We recruited both chronic and acute patients to resemble our hospitalized patients more closely to cancer patients. Then, we selected our study hospitalized patients using stratified random sampling according to a five-year age interval and residential location (living in or outside of the capital city of the province). We excluded potential hospitalized patients that were too ill to answer the questionnaire.

#### Healthy individuals group

Healthy individuals were the companions of chronic disease patients in the same hospitals where our hospitalized patients were recruited. We recruited healthy individuals with a sampling method similar to hospitalized patients.

### Questionnaire and data collection

We confirmed the face and content validity of the questionnaire through several expert opinion meetings. We examined the test-retest reliability of the questionnaire on a convenience sample of 50 addicts who were referred to an addiction treatment center on two occasions separated by a two week-interval. All reliability indices were above 0.7. The questionnaire included questions on opioid use [12] including raw opium [13], Shireh (the condensed extract of remnants of smoked opium)[12] and Sukhteh (the remnants of smoked opium) [14] considered together as opium, and also heroin, Crack of heroin (a heroin-based narcotic used in Iran which is different from the cocaine-based crack used in Western countries) [15], and non-medical morphine, as well as the history of use of other substances, including alcohol and tobacco (cigarettes and hookah). Moreover, it included questions on demographic characteristics, socioeconomic status, job title, diseases, and use of medicines, including drugs containing codeine and morphine during the last two weeks, to differentiate medical opioid use from illegal opioid use.

For hospitalized patients, we recorded the date of their first symptom and disease onset and also their pain status (severe, moderate, or mild) according to their self-reports. Regular substance use was defined as consumption at least once a week for at least a six month period during the subject’s lifetime. Also, we asked participants about the date of their last opioid use to compare their self-reports with urine test results. Although participants were aware of the study hypothesis, they were not aware of these specific hypotheses unless they inquired during the process of obtaining informed consent.

Interviewers with a Bachelor of Arts degree in psychology or social work were specially trained for this study in a two-day workshop to standardize the interviewing process and mitigate inter-rater variability. A detailed questionnaire guideline was used. All the participants were invited for interview in private rooms. We introduced the study to participants; for people who refused to participate, we collected information on age, education level, marital status, and the reasons for refusal using a query form called the refusal form. After the interview, we collected urine samples for laboratory analyses.

### Laboratory tests

In this study, we used two types of urine drug screens tests, urine rapid drug screen (URDS) and immunoassay thin layer chromatography (TLC). The URDS test is one of the most common methods for the initial screening procedure [16]. TLC uses antibodies to detect metabolites or the presence of specific drugs and is considered the gold standard for confirmatory testing. The method can detect small amounts of a substance and is the most sensitive, accurate, and reliable method of testing; conversely, the test is time-consuming, a high level of expertise is required to perform it, and it is costly. Consequently, TLC is usually done only after a positive result is obtained in the URDS test [16].

For the initial screening, we used the URDS test to assess the presence of opioid metabolites, including morphine (cutoff point: 300 ng/ml). The test protocol was provided by the toxicology laboratory of Iranian National Center for Addiction Studies (INCAS). Metabolites of morphine are detectable in the urine, at the utmost, for 72 hours after use. Consequently, TLC tests were used for participants with a positive URDS test result, but who denied opioid use in the questionnaire, either regular use or during the past 72 hours. [17].

### Statistical analyses

We performed descriptive analysis using mean and proportions for quantitative and qualitative variables. We then used chi-squared tests and t-tests to compare the two groups with respect to demographic variables. In order to adjust for the clustering effect of conducting a multicenter study, we performed multilevel logistic regression analysesbut the result was not significantly different from the simple analysis. Therefore, we present the simple analysis results for easier interpretation (S1 and S2 Tables).

We used information on the use of morphine in the past 72 hours and the results of the urine tests. TLC was considered as the gold standard. We compared the sensitivity of self-reported opioid use in the past 72 hours in both groups. As opioid metabolites are detectable in the urine for 72 hours, to calculate the sensitivity, the numerator was the number of participants who consumed opioids during the past 72 hours based on their self-reports, and the denominator was the number of people who consumed opioids in the past 72 hours (sum of self-report and TLC test result).

### Study power

We used a sample size calculation formula for comparing the two proportions. Assuming a 15% difference between proportions, a type one error (α) of 5%, and a design effect of 1.5, about 170 subjects were needed in each group to evaluate the sensitivity of the questionnaire for the evaluation of opioid use with 80% power.

### Ethical considerations

The ethics committee of Tehran University of Medical Sciences approved the study (Ethical code: 27867–142022). All participants provided written informed consent. The data of the participants were handled confidentially. Urine samples were kept anonymous for investigators and the results were not linked to participants’ identities in the dataset. Participants received a small gift after interview.

## Results

### Participant characteristics

Healthy individuals and hospitalized patients were not significantly different regarding the distribution of their demographic characteristics (Table 1). Participation rates were high in both healthy individuals (86%) and hospitalized patients (88%). Non-participants were defined as people who refused to participate after study introduction. About half of the non-participation rates were due to unwillingness to provide urine samples in both groups.

**Table 1:** Comparison of healthy individuals and hospitalized patients according to demographic variables and self-reported regular substance use.

	Healthy Individuals (n = 186)	Hospitalized Patients (n = 178)	P-value
	Mean (SD)	Mean (SD)	
**Age**	53.2 (11.8)	53.1 (12.2)	0.9
	Number (%)	Number (%)	
**Education**			
≤ High school	153 (82.3)	157 (88.2)	0.1
> High school	33 (17.7)	21 (11.8)	
Marital status*			
Single	11 (6)	13 (7.3)	0.6
Married	17 5(94)	165 (92.7)	
**History of regular substance use**			
Opioid use**	36 (19.3)	65 (36.5)	0.001***
Opium (raw opium, Shireh and Sukhteh)	35 (18.8)	64 (36)	0.001***
Crack of heroin	1(0.5)	2 (1.1)	0.5
Heroin	3 (1.6)	1 (0.6)	0.3
Morphine (without prescription)	0	1 (0.6)	0.3
**Alcohol use**	25 (13.4)	30 (16.8)	0.4
**Cigarette use**	82 (44.1)	80 (45.2)	0.8
**Hookah use**	1 3(7)	8 (4.5)	0.3

*Frequency of widowed and divorced people were low, so we considered them as single.

** Opioid use refers to use of raw opium, Shireh (the condensed extract of remnants of smoked opium), Sukhteh (remnants of smoked opium), Crack of heroin (crystalized form of heroin), and morphine (without prescription).

*** significant at the 0.05 level

The median duration of disease in hospitalized patients was 5.5 months (interquartile range: 0.01–3 years). Hospitalized patients were recruited in different wards, including orthopedics (17.4%), endocrinology (16.9%), surgery, (13%), neurology (10%), urology (10%), and other wards (32.7%) including ear, nose & throat, internal medicine, gastroenterology, neurosurgery, infectious, and eye disease wards. Most of the participants had no (43.3%) or mild (27%) pain.

### Prevalence of self-reported opioid use

**Fig 1:**
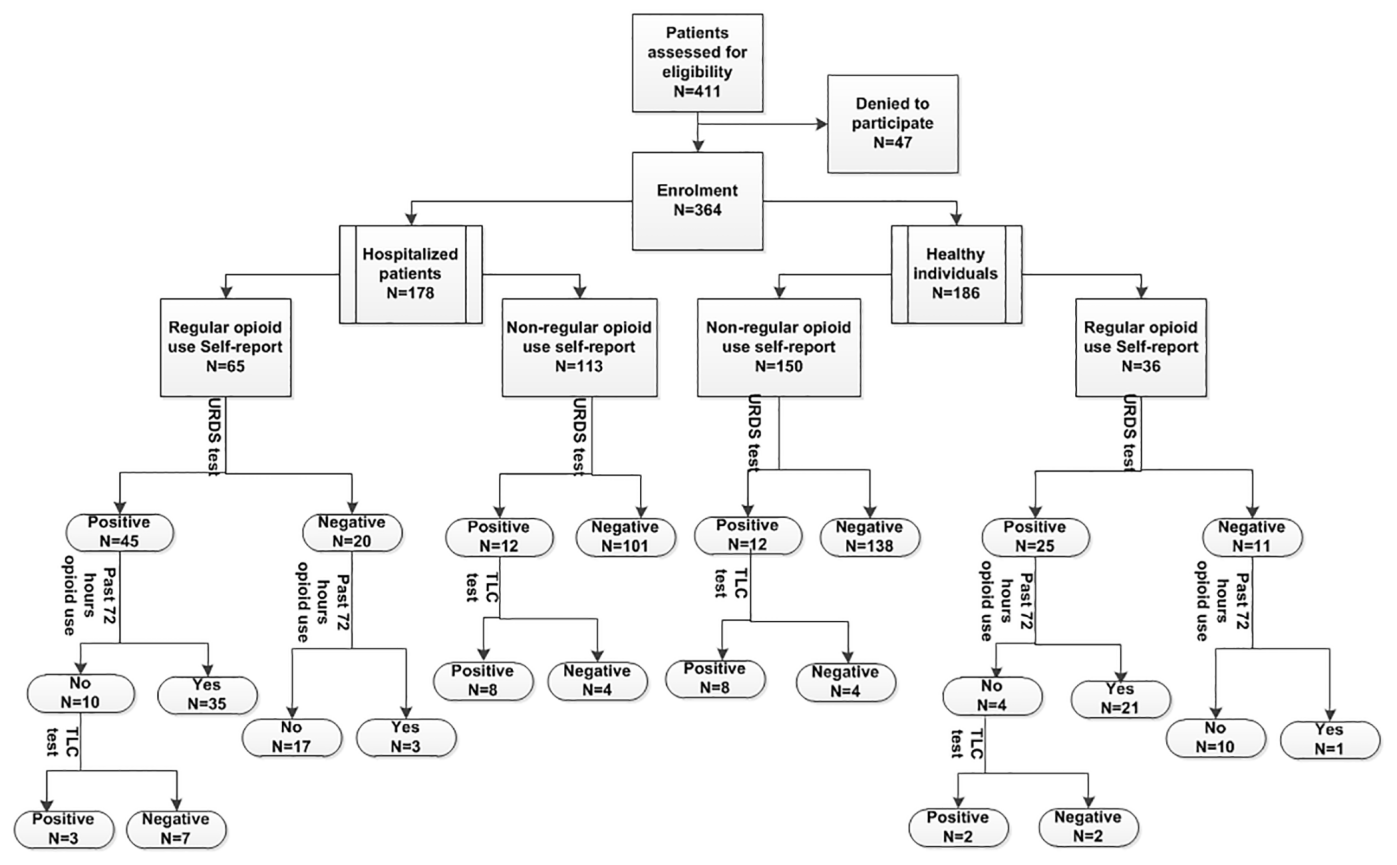
Flowchart of study participation, self-reported opioid use and urine test results in healthy individuals and hospitalized patients.

Self-reported regular opioid use was significantly lower in healthy individuals (19.3%) than hospitalized patients (36.5%) (Table 2). Out of the 186 healthy individuals, 36 reported regular opioid use. Among the 36 healthy individuals who were regular opioid users, 22 (61.1%) reported opioid use during the past 72 hours. Out of 65 hospitalized patients who were regular users of opioids, 38 (57.6%) reported using opioids during the past 72 hours (Fig 1). In addition, about 10 percent of regular opioid users in the hospitalized patients started opioid consumption after the onset of their disease according to their self-reports.

**Table 2:** Comparison of healthy individuals and hospitalized patients in terms of opioid use based on self-report and TLC test results.

Subject	healthy individuals		hospitalized patients		P-value
	N*	n* (%)	N*	n* (%)	
1. Regular opioid use (at least once a week for at least a six month period during the subject’s lifetime) based on self-report**	186	36 (19.3)	178	65 (36.5)	>0.001***
2. consumed opioids during the past 72 hours based on self-report	186	22 (11.8)	178	38 (21.4)	0.01***
3. Positive TLC test results among those who denied opioid use during the past 72 hours	164	10 (6.1)	140	11 (7.9)	0.7
4. Consumed opioids in the past 72 hours (sum of rows 2 and 3)	186	32 (17.2)	178	49 (27.5)	0.02***
5. Sensitivity of self-report in those who consumed opioids during the past 72 hours	32	22 (68.8)	49	38 (77.5)	0.4

* The denominator for the calculation of each row was not the same; we show denominator for each row with the letter N and the numerator with the letter n.

** Opioid use refers to regular use of raw opium, Shireh (the condensed extract of remnants of smoked opium), Sukhteh (remnants of smoked opium), Crack of heroin (crystalized form of heroin), and morphine (without prescription).

*** Significant at the 0.05 level.

### Sensitivity of self-reported opioid use

Among the 36 healthy individuals who reported regular opioid use, the URDS test was positive in 25 (69.4%). Out of the 37 healthy individuals with a positive morphine URDS test result, 16 (43.2%) did not report regular or past 72 hours opioid use during the interview. TLC was positive in 10 of this group (Fig 1).

The URDS for morphine test results were positive for 45 (69%) and negative for 20 (31%) of patients who reported regular opioid use. According to the URDS test results, 22 hospitalized patients did not report opioid use regularly or in the past 72 hours during the interview. We performed the TLC test for these patients. The TLC test result was positive for 11 (50%) of them (Fig 1).

We compared two groups in terms of the sensitivity of their self-reported opioid use during the past 72 hours. According to the URDS and TLC test results, the sensitivity of self-reported opioid use during the past 72 hours was 77% (CI: 65.8% -89.2%) in the hospitalized patients and 69% (CI: 52.8% -84.9%) in the healthy individuals (Table 2).

### Prevalence based on TLC results

In order to estimate the true prevalence of self-reported regular opioid use, we divided this value by the sensitivity of self-reported opioid use within the past 72 hours. True prevalence of regular opioid use according to the sensitivity was estimated to be 28% (CI: 21.6–35) and 47.4% (CI: 39.7–54.8) for healthy individuals and hospitalized patients, respectively. This was significantly lower in healthy individuals than in hospitalized patients (P-value≤0.001).

## Discussion

We conducted a cross-sectional study to measure the sensitivity of self-reported opioid during the past 72 hours use among healthy individuals and hospitalized patients in a case-control study. Regular opioid use was significantly more frequent in hospitalized patients than in healthy individuals, even after correcting for underreporting. However, hospitalized patients and healthy individuals did not differ in terms of assessing the sensitivity of opioid use in the past 72 hours, non-participation rates, and reasons for non-participation.

The prevalence of regular opioid use in hospitalized patients was significantly higher than in other studies [18] and to what is estimated to be the prevalence of regular opioid use in the general population in Iran, namely 15–20% [19, 20]. This may be because opioids are sometimes used as a pain reliever among hospitalized patients, or because long-term opioid use may cause disease. Previous studies have reported statistically significant positive associations between opium use and a higher risk of some of common diseases, such as cardiovascular disease [21, 22], cancer [23], and all-cause mortality[24]. Despite our strict inclusion and exclusion criteria for the selection of hospitalized patients, we cannot rule out that the cause of hospitalization among hospitalized patients was not related to opioid use. Bias by indication could have occurred, as the patients who had chronic disease could use opioids to mitigate pain and discomfort before hospital admission.

The prevalence of regular opioid use in healthy individuals was lower than the hospitalized patients, and closer to the estimated prevalence of regular opioid uses in general population [19, 20]. On the other hand, the sensitivity of assessing opioid use in the past 72 hours was similar in both groups; thus, the lower prevalence of regular opioid use in healthy individuals is less likely due to an underreporting bias.

Previous studies have shown that substance use is more prevalent in people younger than 50 years of age [10]. In this study, we considered the same age distribution for both groups; thus, the differences between healthy individuals and hospitalized patients cannot be attributable to their age distribution differences. Substance use has been reported to be less prevalent among married people and in people with a higher educational level [10]. However, we found no statistically significant difference in marital status or educational level between the two groups.

Moreover, there were no significant differences in participation rates between the two groups in our study. However according to other studies, healthy individuals usually have a lower participation rate [25] and underreport their substance use, as a result of feeling insecure [3, 26]. The high participation rates in our study could be due to the fact that we recruited subjects in hospitals, and because we used well-trained interviewers who were strictly supervised.

According to the current study results, healthy individuals are superior to hospitalized patients as control for conducting case-control study. This is consistent with a study by Shakeri et al [18] on the association between the risk of squamous cell carcinoma of the esophagus and opium use. According to this study, neighborhood controls were superior to hospitalized patients as their prevalence of opium use was more similar to the general population. However, according to our study results, underreporting in both groups was not ignorable and studies on sensitive issues like opium use should be careful about false negative results and correct their estimations for this bias, which could lead to finding a spurious association.

### Strengths and limitations

Cross-validating self-reported opioid use within the past 72 hours with urine test results is strength of the current study. This is the first study in Iran to compare urine test results with self-reported opioid use in the past 72 hours instead of regular opioid use. Moreover, we used well-trained interviewers under strict supervision, resulting in a high participation rate. One of the limitations of the current study was the impossibility of calculating the negative predictive value of self-reported opioid use in the past 72 hours, because TLC tests were too expensive and it was not possible to use them for all participants.

### Conclusions

In conclusion, by gaining the trust of healthy individuals, we observed a comparable self-reporting bias to that of hospitalized patients. The prevalence of self-reported regular opioid use among healthy individuals was similar to the general population. Therefore, recruiting healthy individuals as controls in a trustworthy setting like a hospital could be more suitable in conducting case-control studies on the use of substances like opioids. Clearly, without correction for these differences and biases, the results of studies on opioid use can be misleading.

## Supporting information

S1 Table (DOCX)

S2 Table (DOCX)

S1 Dataset Study dataset. (DTA)

## References

[1] Tourangeau R, Yan T. Sensitive questions in surveys. Psychological bulletin. 2007;133(5):859 17723033 10.1037/0033-2909.133.5.859

[2] Zhao J, Stockwell T, MacDonald S. Non—response bias in alcohol and drug population surveys. Drug and alcohol review. 2009;28(6):648–57. doi: 10.1111/j.1465-3362.2009.00077.x 19930019 10.1111/j.1465-3362.2009.00077.x

[3] Shuster JJ, Cook B. Hospital or population controls: a discussion. Journal of chronic diseases. 1983;36(4):315–6. 6833450 10.1016/0021-9681(83)90116-9

[4] Lewallen S, Courtright P. Epidemiology in practice: case-control studies. Community Eye Health. 1998;11(28):57–8. 17492047 PMC1706071

[5] Schulz KF, Grimes DA. Case-control studies: research in reverse. The Lancet. 2002;359(9304):431–4. 10.1016/S0140-6736(02)07605-5 11844534

[6] Infante-Rivard C. Hospital or population controls for case-control studies of severe childhood diseases? American journal of epidemiology. 2003;157(2):176–82. 12522025 10.1093/aje/kwf174

[7] Grimes DA, Schulz KF. Compared to what? Finding controls for case-control studies. The Lancet. 2005;365(9468):1429–33. 10.1016/S0140-6736(05)66379-9 15836892

[8] Abnet CC, Saadatian-Elahi M, Pourshams A, Boffetta P, Feizzadeh A, Brennan P, et al Reliability and validity of opiate use self-report in a population at high risk for esophageal cancer in Golestan, Iran. Cancer Epidemiology and Prevention Biomarkers. 2004;13(6):1068–70. 15184266

[9] Harrison LD, Martin SS, Enev T, Harrington D. Comparing drug testing and self-report of drug use among youths and young adults in the general population. Rockville, MD: Substance Abuse and Mental Health Services Administration, Office of Applied Studies 2007.

[10] Vandad Sharifi M, Hajebi A, Radgoodarzi R. Twelve-month prevalence and correlates of psychiatric disorders in Iran: The Iranian Mental Health Survey, 2011. Archives of Iranian medicine. 2015;18(2):76 25644794

[11] Najafipour H, Masoomi M, Shahesmaeili A, Haghdoost AA, Afshari M, Nasri HR, et al Effects of opium consumption on coronary artery disease risk factors and oral health: Results of Kerman Coronary Artery Disease Risk factors Study a population-based survey on 5900 subjects aged 15–75 years. International journal of preventive medicine. 2015;6. 10.4103/2008-7802.157470 PMC4455126 26097671

[12] Amin‐Esmaeili M, Rahimi‐Movaghar A, Sharifi V, Hajebi A, Radgoodarzi R, Mojtabai R, et al Epidemiology of illicit drug use disorders in Iran: prevalence, correlates, comorbidity and service utilization results from the Iranian Mental Health Survey. Addiction. 2016;111(10):1836–47. doi: 10.1111/add.13453 27177849 10.1111/add.13453

[13] Nasrollahzadeh D, Kamangar F, Aghcheli K, Sotoudeh M, Islami F, Abnet C, et al Opium, tobacco, and alcohol use in relation to oesophageal squamous cell carcinoma in a high-risk area of Iran. British journal of cancer. 2008;98(11):1857–63. doi: 10.1038/sj.bjc.6604369 18475303 10.1038/sj.bjc.6604369 PMC2410115

[14] Sadjadi A, Marjani H, Semnani S, Nasseri-Moghaddam S. Esophageal cancer in Iran: A review. Middle East J Cancer. 2010;1(1):5–14.

[15] Farhoudian A, Sadeghi M, Khoddami Vishteh HR, Moazen B, Fekri M, Rahimi Movaghar A. Component analysis of Iranian crack; a newly abused narcotic substance in Iran. Iranian journal of pharmaceutical research. 2014;13(1):337–44. 24734089 PMC3985238

[16] Moeller KE, Lee KC, Kissack JC, editors. Urine drug screening: practical guide for clinicians Mayo Clinic Proceedings; 2008: Elsevier. 10.4065/83.1.66 18174009

[17] Matson SC. SUBSTANCE ABUSE AND DEPENDENCE Adolescent Medicine Today. 2011:291.

[18] Shakeri R, Kamangar F, Nasrollahzadeh D, Nouraie M, Khademi H, Etemadi A, et al Is opium a real risk factor for esophageal cancer or just a methodological artifact? Hospital and neighborhood controls in case-control studies. PloS one. 2012;7(3). 10.1371/journal.pone.0032711 PMC3291619 22396792

[19] Nakhaee N, Divsalar K, Meimandi MS, Dabiri S. Estimating the prevalence of opiates use by unlinked anonymous urine drug testing: a pilot study in Iran. Substance use & misuse. 2008;43(3–4):513–20. 18365947 10.1080/10826080701772348

[20] Ziaaddini H, Ziaaddini MR. The household survey of drug abuse in Kerman, Iran. Journal of Applied Sciences. 2005;5(2):380–2.

[21] Sadeghian S, Darvish S, Davoodi G, Salarifar M, Mahmoodian M, Fallah N, et al The association of opium with coronary artery disease. European Journal of Cardiovascular Prevention & Rehabilitation. 2007;14(5):715–7. 17925633 10.1097/HJR.0b013e328045c4e9

[22] Sadeghian S, Graili P, Salarifar M, Karimi AA, Darvish S, Abbasi SH. Opium consumption in men and diabetes mellitus in women are the most important risk factors of premature coronary artery disease in Iran. International journal of cardiology. 2010;141(1):116–8. doi: 10.1016/j.ijcard.2008.11.063 19346018 10.1016/j.ijcard.2008.11.063

[23] Kamangar F, Shakeri R, Malekzadeh R, Islami F. Opium use: an emerging risk factor for cancer? The Lancet Oncology. 2014;15(2):e69–e77. doi: 10.1016/S1470-2045(13)70550-3 24480557 10.1016/S1470-2045(13)70550-3

[24] Khademi H, Malekzadeh R, Pourshams A, Jafari E, Salahi R, Semnani S, et al Opium use and mortality in Golestan Cohort Study: prospective cohort study of 50 000 adults in Iran. Bmj. 2012;344:e2502 doi: 10.1136/bmj.e2502 22511302 10.1136/bmj.e2502 PMC3328545

[25] Stavraky KM, Clarke EA. Hospital or population controls? An unanswered question. Journal of chronic diseases. 1983;36(4):301–7. 6833447 10.1016/0021-9681(83)90113-3

[26] Coughlin SS. Recall bias in epidemiologic studies. Journal of clinical epidemiology. 1990;43(1):87–91. 2319285 10.1016/0895-4356(90)90060-3

