# Correction: Sensitivity of self-reported opioid use in case-control studies: Healthy individuals versus hospitalized patients

**DOI:** 10.1371/journal.pone.0192814

**Published:** 2018-02-08

**Authors:** Hamideh Rashidian, Maryam Hadji, Maryam Marzban, Mahin Gholipour, Afarin Rahimi-Movaghar, Farin Kamangar, Reza Malekzadeh, Elisabete Weiderpass, Abbas Rezaianzadeh, Abdolvahab Moradi, Nima Babhadi-Ashar, Reza Ghiasvand, Hossein Khavari-Daneshvar, Ali Akbar Haghdoost, Kazem Zendehdel

The affiliation for the third author is incorrect. Maryam Marzban is not affiliated with #3 but with the Department of Public Health, School of Public Health, Bushehr University of Medical Science, Bushehr, Iran.
