# Correction: Sensitivity of self-reported opioid use in case-control studies: Healthy individuals versus hospitalized patients

**DOI:** 10.1371/journal.pone.0257180

**Published:** 2021-09-02

**Authors:** Hamideh Rashidian, Maryam Hadji, Maryam Marzban, Mahin Gholipour, Afarin Rahimi-Movaghar, Farin Kamangar, Reza Malekzadeh, Elisabete Weiderpass, Abbas Rezaianzadeh, Abdolvahab Moradi, Nima Babhadi-Ashar, Reza Ghiasvand, Hossein Khavari-Daneshvar, Ali Akbar Haghdoost, Kazem Zendehdel

There are errors in the Funding statement. The correct Funding statement is as follows: This study was funded by the National Institute for Medical Research Development (NIMAD) (grant number: 940045).
