# Supplementary material for: Sensitivity of self-reported opioid use in case-control studies: Healthy individuals versus hospitalized patients
Source: PLoS One. 2017 Aug 30;12(8):e0183017. doi: 10.1371/journal.pone.0183017 (PMC5576653; doi:10.1371/journal.pone.0183017)
Supplement: S1 Table — (DOCX) [file pone.0183017.s001.docx]

**S1 Table:** Comparison of healthy individuals and hospitalized patients according to demographic variables and self-reported regular substance use

| **Independent variables** | **OR (95%CI)** |  | **se** | **P- value** |
| --- | --- | --- | --- | --- |
| **Age** | 1.0(1.0-1.0) |  | 0.0 | 0.9 |
| **Education** | 0.6(0.3-1.1) |  | 0.2 | 0.1 |
| **Marital status*** | 0.7(0.3-1.7) |  | 0.3 | 0.5 |
| **History of Regular substance use** |  |  |  |  |
| **Opioid use**** | 0.9(0.4-1.4) |  | 0.3 | ***>0.001 |
| Opium (raw opium, Shireh and Sukhteh) | 2.5(1.5-4.1) |  | 0.6 | ***>0.001 |
| Crack of Heroin | 2.1(0.2-23.4) |  | 2.6 | 0.5 |
| Heroin | 0.3(0.0-3.3) |  | 0.4 | 0.4 |
| Morphine (without prescription) | 0.0 |  | 0.0 | 1.0 |
| **Alcohol use** | 0.0 |  | 0.0 | 1.0 |
| **Cigarette use** | 1.0(0.7-1.6) |  | 0.2 | 0.8 |
| **Hookah use** | 0.6(0.3-1.6) |  | 0.3 | 0.3 |

*Frequency of widowed and divorced people was low, so we considered them as single.

** Opioid use refers to use of raw opium, Shireh (the condensed extract of remnants of smoked opium), Sukhteh (remnants of smoked opium), Crack of heroin (crystalized form of heroin), and morphine (without prescription)

*** Significant at the 0.05 level
