# Supplementary material for: Sensitivity of self-reported opioid use in case-control studies: Healthy individuals versus hospitalized patients
Source: PLoS One. 2017 Aug 30;12(8):e0183017. doi: 10.1371/journal.pone.0183017 (PMC5576653; doi:10.1371/journal.pone.0183017)
Supplement: S2 Table — (DOCX) [file pone.0183017.s002.docx]

**S2 Table:** Comparison of healthy individuals and hospitalized patients in terms of opioid use based on their self-report, urine rapid drug screen test and thin layer chromatography results

| **Subject** | **OR (95%CI)** | | | | **se** | **P- value** |
| --- | --- | --- | --- | --- | --- | --- |
| 1. Regular opioid use (at least once a week during life time for at least a six months period) based on self-report* | 2.5(1.5-4.0) |  |  | 0.6 | | >0.001** |
| 2. consumed opioid during the past 72 hours based on self-report | 2.1(1.2-3.7) |  |  | 0.6 | | 0.01** |
| 3. Positive test results among those who denied opioid use during past 72 hours | 1.3(0.5-3.2) |  |  | 0.6 | | 0.5 |
| 4. consumed opioid in the past 72 hours (sum of rows 2 and 3) | 1.8(1.1-3.0) |  |  | 0.5 | | 0.02** |
| 5. sensitivity of self-report in those who consumed opioid during the past 72 hours | 1.7(0.6-4.8) |  |  | 0.9 | | 0.3 |

* Opioid use refers to regular use of raw opium, Shireh (the condensed extract of remnants of smoked opium), Sukhteh (remnants of smoked opium), Crack of heroin (crystalized form of heroin), and morphine (without prescription)

**significant at the 0.05 level
